# Guidance and examination by ultrasound versus landmark and radiographic method for placement of subclavian central venous catheters: study protocol for a randomized controlled trial

**DOI:** 10.1186/1745-6215-15-175

**Published:** 2014-05-20

**Authors:** Sébastien Perbet, Bruno Pereira, Florian Grimaldi, Christian Dualé, Jean-Etienne Bazin, Jean-Michel Constantin

**Affiliations:** 1 Department of Anesthesiology and Critical Care Medicine, CHU Estaing, CHU Clermont-Ferrand, Intensive Care Unit, F-63000 Clermont-Ferrand, France; 2 Université d’Auvergne, D2R2, EA-7281, Faculty of Medicine, 28 Place Henri Dunant, F-63001 Clermont-Ferrand, France; 3 CHU Clermont-Ferrand, Biostatistics Unit, DRCI, CHU Gabriel-Montpied, F-63003 Clermont-Ferrand, France; 4 CHU Clermont-Ferrand, Centre d’Investigations cliniques, CHU Gabriel-Montpied, F-63003 Clermont-Ferrand, France

**Keywords:** central venous catheter, subclavian vein, ultrasound guidance, pulmonary ultrasound, cardiac ultrasound, contrast test

## Abstract

**Background:**

Central venous catheters play an important role in patient care. Real-time ultrasound-guided subclavian central venous (SCV) cannulation may reduce the incidence of complications and the time between skin penetration and the aspiration of venous blood into the syringe. Ultrasonic diagnosis of catheter misplacement and pneumothorax related to central venous catheterization is rapid and accurate. It is unclear, however, whether ultrasound real-time guidance and examination can reduce procedure times and complication rates when compared with landmark guidance and radiographic examination for SCV catheterization.

**Methods/Design:**

The Subclavian Central Venous Catheters Guidance and Examination by UltraSound (SUBGEUS) study is an investigator-initiated single center, randomized, controlled two-arm trial. Three hundred patients undergoing SCV catheter placement will be randomized to ultrasound real-time guidance and examination or landmark guidance and radiographic examination. The primary outcome is the time between the beginning of the procedure and control of the catheter. Secondary outcomes include the times required for the six components of the total procedure, the occurrence of complications (pneumothorax, hemothorax, or misplacement), failure of the technique and occurrence of central venous catheter infections.

**Discussion:**

The SUBGEUS trial is the first randomized controlled study to investigate whether ultrasound real-time guidance and examination for SCV catheter placement reduces all procedure times and the rate of complications.

**Trial registration:**

ClinicalTrials.gov Identifier: NCT01888094

## Background

Central venous catheters play an important role in patient care, especially in the intensive care unit (ICU). However, their use is associated with various complications, which occur more frequently when catheters are placed through routes involving the subclavian vein than through routes involving the internal jugular and femoral veins [1,2]. Nevertheless, the subclavian vein route is associated with lower rates of infection and thrombosis and remains the route of choice in the ICU [3,4]. Real-time ultrasound-guided central venous cannulation results in a lower technical failure rate (overall and on first attempt), faster access, and a reduction in mechanical complications, although this has been validated mainly for the internal jugular vein [5,6]. One study reported that complications and failure rates of subclavian central venous (SCV) catheterization were lower for real-time ultrasound-assisted vein location [7]. However, the only temporal parameter recorded was the time between penetration of the skin and aspiration of venous blood into the syringe; thus, the time required for the entire procedure has never been evaluated. Ultrasonic diagnosis of catheter misplacement and pneumothorax related to central venous catheterization was reported to be a rapid and accurate method that can be easily performed by ICU physicians [8].

The Subclavian Central Venous Catheters Guidance and Examination by UltraSound (SUBGEUS) study aims to compare the complete time required for the procedure and the rate of complications of a global ultrasound method and a standard (landmark and radiographic) method used in the routine cannulation of the subclavian vein in patients requiring a central line.

## Methods/Design

### Objectives and design

The SUBGEUS study is an investigator-initiated, single center, randomized, controlled trial approved by the Institutional Review Board of Clermont-Ferrand, France (AU-1000, Eudra-CT 2012-A01188-35). Informed consent is obtained from each participant. The SUBGEUS study is conducted in accordance with the Declaration of Helsinki and was registered on June 25, 2013, on http://clinicaltrials.gov/ as trial NCT01888094.

### CONSORT diagram

**Figure 1:**
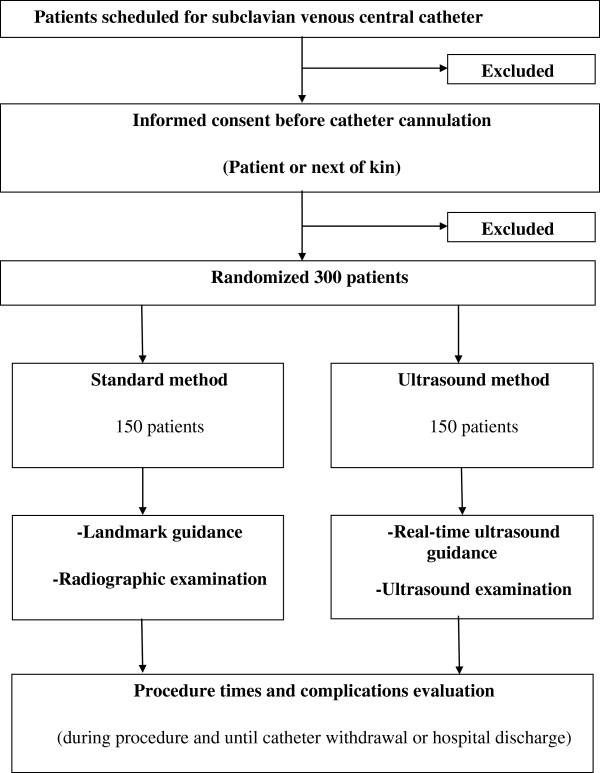
CONSORT diagram of the SUBGEUS trial.

Figure 1 shows the CONSORT diagram of the SUBGEUS trial.

### Study population

Investigators screen consecutive patients who are admitted to the ICU and require a central venous line. Inclusion criteria are requirement for SCV catheterization, age >18 years and informed consent from the patient or his/her next-of-kin. Exclusion criteria are patient refusal, femoral or internal jugular catheterization, and impossibility of obtaining good echogenicity.

All operators must have at least three years of experience with subclavian catheter procedures and must have completed at least five ultrasound procedures.

### Randomization

Patients eligible for inclusion are randomized 1:1 in fixed blocks of 10, without stratification, by a computerized number generator list provided by a statistician not involved in the determination of eligibility or in the assessment of outcomes. All assignments are made through a dedicated, password protected, SSL-encrypted website. Randomized patients are given a number corresponding to a «SUBGEUS Number».

### Study protocol

Patients are randomized to a ‘real-time ultrasound guidance and examination’ method or to a ‘standard’ (landmark and radiographic) method, with study treatment beginning within one hour of randomization.

The predefined length of insertion is based on patient height and calculated as: height/10 - 2 cm for right subclavian vein sites and height/10 + 2 cm for left subclavian vein sites [9]. Patients are placed in the Trendelenburg position. The operator will evaluate the likelihood of difficult cannulation, based on various patients factors, including obesity (body mass index >30 kg/m^2^), coagulation disorders, short neck, acute respiratory failure, agitation, and history of SCV cannulation.

Before starting the procedure, the operating physician uses maximal sterile-barrier precautions, including a mask, a cap, a sterile gown and gloves and handwashing.

The various procedure times, T0 to T5, are defined as:

T0: Start of the procedure, defined as when the physician begins skin disinfection with 2% chlorhexidine. Large sterile drapes are set. If necessary, a local anesthetic (1% lidocaine solution) is applied with a 22-gauge needle. Ultrasound guided determination of the puncture site is identified in patients randomized to the ultrasound group.

T1: Skin puncture.

T2: Return of venous blood into the syringe attached to the needle, followed by correct insertion of the guide according to Seldinger’s technique. In case of uncorrected insertion of the guide, a new site is punctured, with T2 defined as the time of return of venous blood from the new puncture, followed by correct insertion of the guide.

T3: Application of dressing after catheter insertion, purge and fixation. Lines of perfusion are positioned. Ultrasound examinations are started or the radiology technician in the radiology department is contacted. The chest radiograph device is located in the ICU.

T4: The catheter is checked for placement and complications. A chest radiograph is taken and interpreted or the patient has been assessed by ultrasound examination, depending on randomization. If there is no evidence of complications or misplacement, then T4 = T5. If there are complications or misplacement, corrections are made, including a chest tube for pneumothorax, and withdrawal or repositioning if the catheter is misplaced.

T5: End of the procedure, when the catheter is accurately inserted and controlled.

Complications recorded include arterial puncture, pneumothorax, hemothorax, and misplacement (contralateral subclavian or jugular route; outside the superior vena cava or the right atrium).

### Landmark method

The ipsilateral anterosuperior region of the chest is shaved if necessary and skin disinfected with 2% chlorhexidine. If necessary, a local anesthetic (1% lidocaine solution) is applied. The needle is inserted 1 cm inferior and 1 cm lateral to the junction of the middle and medial thirds of the clavicle. Patients for whom cannulation is not possible with the landmark method will undergo the procedure under ultrasound guidance on the ipsilateral side. If highlighted thrombosis occurs, the patient will be withdrawn from the study and will undergo the procedure under ultrasound guidance on the contralateral side.

Correct placement of the tip of the catheter after the procedure is defined as <2.9 cm caudal to the right tracheobronchial angle to avoid intracardiac placement, as previously described [10].

### Ultrasound method

The area is prepared (skin disinfection with 2% chlorhexidine, local anesthesia with lidocaine 1%) and draped sterilely as described above. In addition, the ipsilateral internal jugular and contralateral subclavian areas are prepared in case of catheter misplacements for proper ultrasound-guided repositioning. An Envisor or CX-50 ultrasound machine (Philips, Andover, MA, USA) equipped with a high-resolution 7.5-MHz transducer is used. The transducer is covered with ultrasonic gel and wrapped in an intraoperative sterile sheath (CIV-Flex^TM^ Transducer cover, 14 × 147 cm; CIVCO, Kalona, IA, USA).

**Figure 2:**
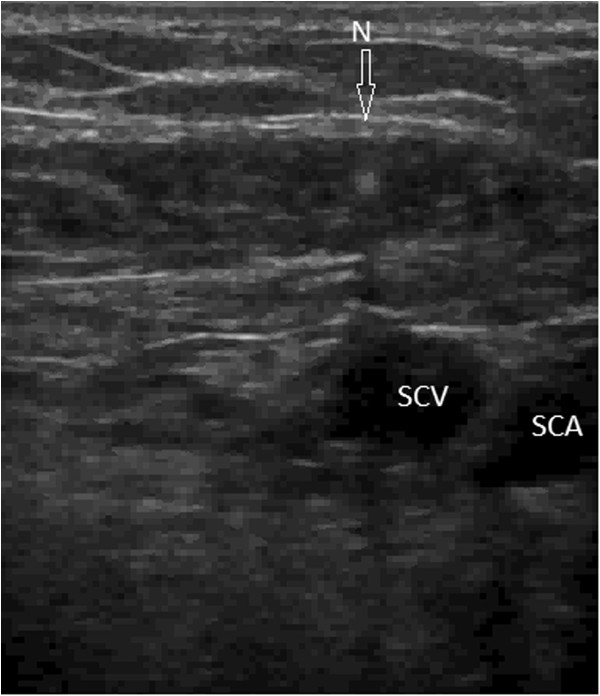
**Out-of-plane view (short axis) of the needle entering the subclavian vein with the acoustic shadow in an infraclavicular approach.** N, needle; SCA, subclavian artery; SCV, subclavian vein.

**Figure 3:**
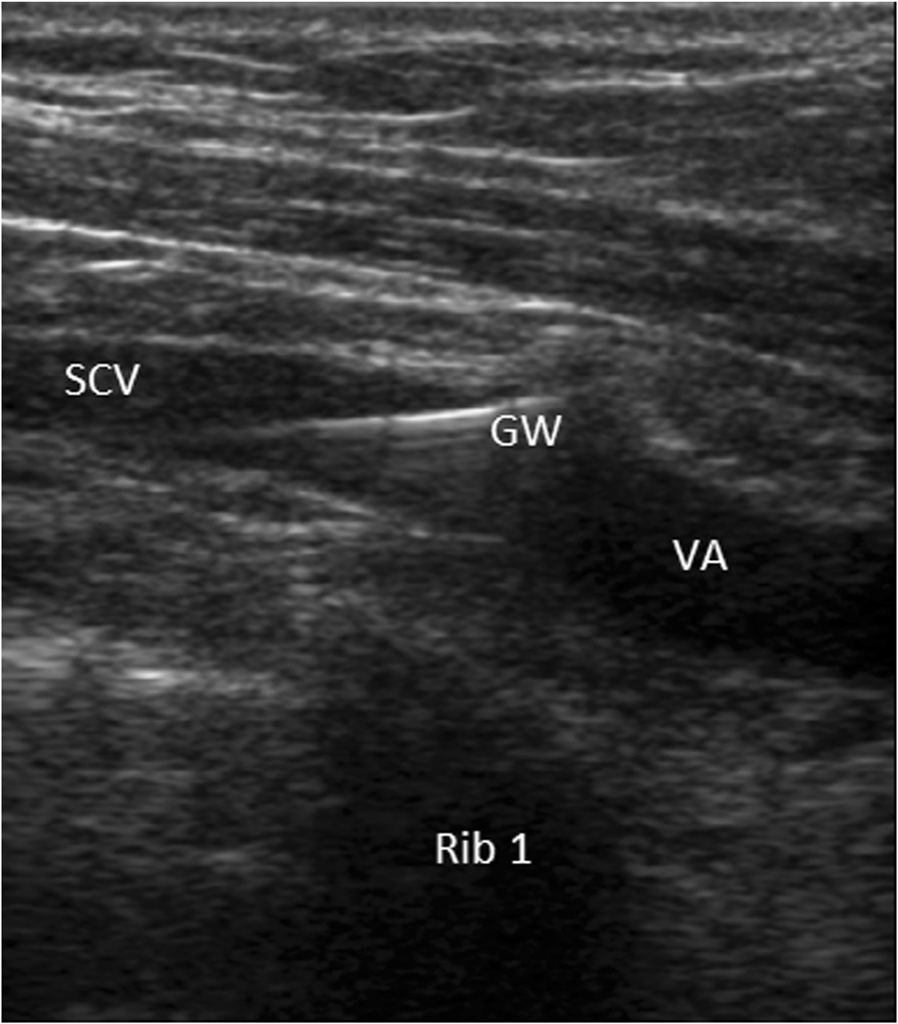
**In-plane view (long axis) of guidewire placement in the subclavian vein using an infraclavicular approach.** AV, axillary vein; GW, guidewire; Rib 1, acoustic shadow on the first rib; SCV, subclavian vein.

The ultrasound method applied for SCV catheterization is a four-step procedure:

1. Preprocedural ultrasound scanning, through an infraclavicular views, is performed to measure the depth and caliber of the subclavian vein and to evaluate its patency. Furthermore, it is used to identify adjacent structures. The infraclavicular approach is also chosen for the area of puncture; if this approach makes evaluation difficult, the supraclavicular approach will be considered [11].

2. An infraclavicular approaches, as described previously [7,12-14], is utilized to compare the ultrasound and landmark methods. Although we use a high-frequency transducer, which applies tissue harmonic imaging, depicting two-dimensional views of the vein is difficult. Physicians use sonoanatomic landmarks such as the acoustic shadows of the underlying first thoracic rib and of the sternum to select an area of interest. The needle is inserted at the midclavicular line or more laterally, 1 to 3 cm under the clavicle. Under ultrasound guidance, the needle is advanced through the area between the clavicle anteriorly and the first rib posteriorly. The transducer is maneuvered to depict the subclavian vein, either the longitudinal (in-plane) or the transverse (out-of-plane) axis, and to achieve an optimum plane of catheterization (Figures 2 and 3). Doppler techniques are also used to confirm the two-dimensional findings.

3. The needle is advanced slowly, so that its trajectory and/or tip can be detected superficially, toward the lumen of the vein on the longitudinal axis, while being purposefully directed toward the acoustic shadow of the thoracic rib underneath, thus minimizing the risk of damaging the pleurae and lung if transfixion of the vein is inevitable. Therefore, the needle enters the lumen of the vessel at the level of the subclavian vein, depending on the angle of penetration and the depth of the vein from the skin surface. In addition, the course of the needle is dependent on adjustments performed by the operator to visualize its trajectory on the longitudinal axis. Hence, the angle of penetration and the course of the needle can be modified in each patient during catheterization, making this technique largely operator-dependent. If a supraclavicular approach is used, the needle is inserted with the probe placed in the supraclavicular fossa, just lateral to the clavicular head of the sternocleidomastoid muscle in a long or short axis. The needle enters the subclavian vein before its confluence with the internal jugular vein. 4) The guidewire is advanced according to Seldinger’s technique; thereafter, the internal jugular veins and the contralateral subclavian vein are scanned to identify possible misplacements, allowing the catheter to be repositioned under ultrasound guidance.

Postprocedural ultrasound examination includes detection of:

1. Pneumothorax, as determined from lung-sliding, comet-tail artifacts, lung-point and lung pulse. Between two ribs (identifiable by their acoustic shadow), the interface between the thoracic wall and the lung consists of a hyperechogenic line that represents both the visceral and parietal pleura. Lung-sliding is a to-and-fro movement of this hyperechogenic line, which follows respiratory movements [15]. Comet-tail artifacts are grossly vertical artifacts spreading from the hyperechogenic line to the inferior edge of the screen [16]. The lung pulse refers to the subtle rhythmic movement of the visceral upon the parietal pleura with cardiac oscillations [17]. The lung point refers to the depiction of the typical pattern of pneumothorax, which is simply the absence of any sliding or moving B-lines at a physical location where this pattern consistently transitions into an area of sliding, representing the physical limit of pneumothorax as mapped on the chest wall [18].

An algorithm for diagnosing pneumothorax was recently proposed, based on the sonographic signs of pneumothorax [19]:

absence of lung sliding

absence of B-lines

presence of lung point(s)

absence of lung pulse.

2. Hemothorax with absence of pleural effusion at the pleural dead-end.

3. Misplaced catheters, either at an aberrant or too distal position. An aberrant position is defined as catheter-tip visualization: a) in the ipsilateral internal jugular vein or contralateral subclavian vein, or b) in the contralateral internal jugular vein. A too distal position is defined as visualization of the catheter tip in the intracardiac position - intra-atrial or intraventricular - or in the inferior vena cava.

4. Postprocedural ultrasound examination also includes contrast tests: saline transthoracic contrast echocardiography performed to confirm appropriate placement consists of injecting 10 ml of normal saline in a 10-ml syringe and checking the induced contrast in the right atrium with linear flow coming from superior vena cava within 2 sec [20,21].

Patients in the ultrasound group also undergo a chest x-ray after the procedure to confirm the absence of pneumothorax or misplacement.

### Study endpoints

The primary endpoint is the procedure time between the beginning of the procedure (T0) and when the catheter is controlled (T4).

The secondary outcomes are the procedure times (T0 to T5) of the two strategies, rates of per procedure complications (pneumothorax, hemothorax, and misplacement), failure of the technique and central venous catheter infection (catheter-related sepsis without bacteremia or catheter-related bloodstream infections). Catheter-related sepsis without bacteremia and catheter-related bloodstream infections are described below:

1. Catheter-related sepsis without bacteremia is defined according to the combination of 1) fever (body temperature ≥38.5°C) or hypothermia (body temperature ≤36.5°C), 2) catheter colonization, 3) regression of fever or hypothermia within the 48 h following catheter removal and with no change in antimicrobial therapy, or presence of pus at the catheter insertion site, and 4) no other identified sources of infection.

2. Catheter-related bloodstream infections are defined as the combination of 1) fever (body temperature ≥38.5°C) or hypothermia (body temperature ≤36.5°C), 2) one or more positive peripheral blood cultures drawn 48 h before or after catheter withdrawal, 3) isolation of the same organism (same species and same susceptibility pattern) from the colonized catheter or from the catheter insertion site, or a blood-culture differential time-to-positivity of 2 h or longer, and 4) no apparent source of bacteremia other than the catheter. In patients with positive coagulase negative staphylococci bacteremia, at least two positive cultures from separate blood samples are required.

### Follow-up

The following variables are collected:

Pre-randomization and during baseline assessment - gender, age height, weight, cardiac status (hypertension, cardiac failure, and ischemic heart failure), respiratory status (history of chronic obstructive pulmonary disease, asthma status, and long-term home oxygen), smoking status, alcohol status, history of diabetes mellitus, IGS 2, SOFA, indication for the SCV catheter and blood parameters (WBC count, hemoglobin, prothrombin time, fibrinogen, urea, and creatinine).

During the catheter procedure - highlighted thrombosis, the various procedure times (T0 to T5), number of punctures, number of catheter kits used, change in cardiac rhythm during wire insertion, use of the supra- or infra-clavicular approach, out-of-plane or in-plane ultrasound approach, depth of the subclavian vein, difficulty of subclavian vein visualization (very easy, easy, intermediate, difficult, or impossible), switch of the puncture site, operator or randomization group, arterial puncture, absence of internal jugular or contralateral subclavian placement, control of contrast test, absence of pneumothorax, and hemothorax.

After the procedure - duration of catheterization, occurrence of catheter infection.

### Sample size estimation, protocol drop-out and suspension

Studies have shown that ultrasound can detect the absence of complications and the correct positioning of the catheter and can reduce the processing time of the audit report on chest radiographs [8,22]. The standard deviation of the processing time varies between 5 min and 25 min. In this study, 2 × 133 patients are needed to detect a 10 min (standard deviation equals 25 min) difference in the primary outcome, at a two-sided α level of 0.05 and a statistical power of 90%. It was estimated that 10% of patients would cross-over or drop-out, resulting in a total of 300 patients, 150 in each group. An interim analysis is planned after enrollment of the first 82 (1/4 of inclusions) patients to review data relating to patient safety and quality of trial conduct (software East (Cambridge, MA, USA, http://www.cytel.com), Kim-DeMets method with Pocock boundary). The committee monitoring data and safety will recommend stopping the trial if its continuation will compromise patient safety, defined as a between-group difference in serious adverse events.

If ultrasound highlights a thrombosis, the other subclavian vein will be used for cannulation. If cannulation of the subclavian vein is impossible, the patient will be excluded from the study and cannulation will be attempted using the internal jugular or femoral vein. These patients will be analyzed according to their originally assigned group on an intention-to-treat (ITT) basis.

### Statistical analysis

Statistical analyses will be performed on ITT populations. Student’s unpaired *t*-test or the Mann-Whitney test (if appropriate) will be used for primary outcome analysis. The Shapiro-Wilk test will be used to assess normality, and the Fisher-Snedecor test to assess homoscedasticity. Analysis will be adjusted by random effects linear regression to 1) take into account adjustments for possibly confounding covariates selected according to clinical relevance (for example, history of venous central catheter procedures, failure rate of the technique) and 2) consider variations within and between centers and operators. All effect sizes will be reported with 95% confidence intervals. Other continuous variables will be analyzed identically. Categorical parameters will be compared in the randomized groups using unadjusted chi-square or Fisher’s exact test. Analyses will be adjusted using the previously described adjustment variables in a logistic regression model. If the frequency of missing data is >5%, an additional analysis will be performed using the multiple imputation method (software Stata command [23]). All analyses will be performed using Stata statistical software, release 12 (StataCorp, College Station, TX, USA).

### Data handling and record keeping

Data will be handled according to French law. All original records (including consent forms, CRFs and relevant correspondence) will be archived at the trial site for 15 years. The clean database file will be made anonymous and maintained for 15 years.

### Study organization

Financial support is provided by a research grant from the University Hospital of Clermont-Ferrand. The study is promoted by Clermont-Ferrand University Hospital.

The independent Data and Safety Monitoring Board (DSMB) ensures that the trial is in accordance with the Declaration of Helsinki, monitors patient safety and reviews safety issues as the study progresses. Serious adverse events and unexpected related or possibly related serious events are reported blinded to the DSMB within 24 hours and 7 days, respectively.

## Discussion

It has become clear that ultrasound approaches have potential applicability in central venous catheterization. All previous reports, however, have evaluated parts of the procedure time during real-time ultrasound guidance [7]. The times of preparation of the transducer, which is covered with ultrasonic gel and wrapped in an intraoperative sterile sheath, and of the ultrasound guidance itself have not been yet evaluated. These may limit the findings showing that generalized ultrasound approaches reduce the time of catheter insertion. The SUBGEUS trial is the first randomized controlled study to investigate whether ultrasound real-time guidance and examination for SCV catheter procedure improves all procedure times.

The primary end-point of the trial is the total procedure time, from the beginning of procedure until the catheter is controlled. This time may be regarded as being more representative of the procedure time than the time between penetration of skin and aspiration of venous blood into the syringe.

The time to process audit reports on chest radiographs is reported to be very long, around 80 minutes, and may depend on the center. In contrast, the time required for ultrasound examination was much shorter (6.8 ± 3.5 min) [8]. A prior evaluation in our institution showed that the median processing time was 20 min and ranged from 5 min to 25 min. The device used for chest radiography is located in our ICU, and the assistant radiologist must simply be called to take the radiograph with this device. Potentially, it may be difficult to generalize our results concerning this part of procedure.

Nevertheless, immediate ultrasound determination of the absence of misplacement or complications could allow the very early use of SCV catheters for multidrug perfusion in critically ill patients. The few minutes saved may represent a potential improvement of vital functions in the emergency room.

In conclusion, the SUBGEUS trial is an investigator-initiated pragmatic randomized controlled trial powered to test the hypothesis that a complete ultrasound approach (real-time guidance and examination) reduces the procedure time, from the beginning of the procedure to the end of checking the catheter. The SUBGEUS trial also determines the impact of the ultrasound approach on most procedure times and on the occurrence of complications.

## Trial status

The trial is ongoing and is actively enrolling patients.

## Abbreviations

DSMB: Data and Safety Monitoring Board; ICU: intensive care unit; ITT: intention-to-treat; SUBGEUS: Subclavian Central Venous Catheters Guidance and Examination by UltraSound.

## Competing interests

The authors declare that they have no competing interests.

## Authors’ contributions

SP prepared the initial drafts of the manuscript and the final version. BP planned the statistical analysis and revised the manuscript. SP, BP, CD, FG, JEB, and JMC designed the study and reviewed the initial drafts of the manuscript. All authors approved the final version of the manuscript. All authors read and approved the final manuscript.
